# The complete mitochondrial genome of *Scorpiops tibetanus* (Scorpionida; Euscorpiidae Laurie)

**DOI:** 10.1080/23802359.2020.1848475

**Published:** 2021-02-05

**Authors:** Heng Zheng, Li-Bo Xiang

**Affiliations:** a Institute of plant protection and soil fertilizer, Hubei Academy of Agricultural Sciences, Wuhan, Hubei, China; b College of science, Tibet University, Lhasa, Tibet, China

**Keywords:** *Scorpiops tibetanus*, mitochondrial genome, phylogenetic analysis

## Abstract

This study is the first to sequence and analyze the mitochondrial genome of *Scorpiops tibetanus* . The results showed that the total length of mitochondrial genome of *Scorpiops tibetanus* was 14,825 bp, including 13 protein-coding genes, 22 tRNAs, 2 rRNAs, and 1 control region. According to the complete mitochondrial genome, the phylogenetic tree was established by the *Scorpiops tibetanus* and six closely related species. Phylogenetic tree analysis showed that the *Scorpiops tibetanus* is sister species to *Heterometrus longimanus*. This study provides theoretical references for further exploring the genetic diversity and phylogeny of scorpion.

*Scorpiops tibetanus* Hirst, 1911 is an important biological resource, it belongs to the Arthropoda, Arachricla, Scorpionida, Euscorpiidae Laurie, *Scorpiops tibetanus,* which is endemic to Tibet Autonomous Region of China. It lives in the Qinghai Tibet plateau where the ecological environment is fragile. The *Scorpiops tibetanus* venom contains new antimicrobial peptides stct1 (Yuan et al. 2010) and stct2 (Cao et al. 2012). In addition, Tibetanin, a unique toxin, is detected in the *Scorpiops tibetanus* (Ma et al. 2010). At present, researches on the *Scorpiops tibetanus,* especially the genetic information are rarely. In this study, we sequenced the complete mitogenome of *Scorpiops tibetanus* to further understanding the mitogenomic characteristics and explore the phylogenetic relationships. The *Scorpiops tibetanus* was collected from Lhasa (E91.181607,N29.645796), Tibet Autonomous Region, China. The specimen was stored in the herbarium of Institute of Plant Protection, Soil and Fertilizer, Hubei Academy of Agricultural Sciences (specimen voucher：20200701). As we know, this is the first study to sequence and analyze the mitochondrial genome of *Scorpiops tibetanus*.

Using Hipure Universal DNA Kit (Genepianeer Biotechnologies Co., Ltd.) to extract DNA from *Scorpiops tibetanus*. After the sample DNA was qualified, DNA was fragmented by ultrasound. The fragment DNA was purified, the terminal was repaired, the 3 'end was added with A, the DNA sequencing was joined, and the fragment size was selected by agarose gel electrophoresis. VAHTS Universal DNA Library Prep Kit for Illumina V3 (Vazyme Biotech Co., Ltd.) was used to construct the library. The library template size was about 300 bp. Sequencing was performed on Illumina novaseq platform. Fastp (V 0.20.0) is used to filter and prune the original sequence, and the Bowtie2 (V2.2.4) compared the sequence with the self-built animal mitochondrial database, assembled the sequence on the pair with Spades (V3.13.1) to get contig, sSpace (V2.0) for scaffold connection, and gap filter (V2.1.1) for hole filling. The annotation results were compared with six closely related species, and the final annotation results were obtained after manual correction. The obtained sequence was annotated by using Mitos2 online website, and the final annotation result was obtained after checking and modifying. After assembly and annotation, the complete mitochondrial genome of *Scorpiops tibetanus* was submitted to GenBank with the accession number of MT903349.

The results showed that the complete mitochondrial genome of *Scorpiops tibetanus* was 14,825 bp, containing 13 PCGs, 22 tRNAs, 2 rRNAs, and 1 control region, which was similar to other reported scorpions (Choi et al. 2007; Sonia et al. 2005). The base composition of the H chain, A, G, C and T was 30.27, 41.50, 16.15, and 12.08%, respectively. Among the mitochondrial PCGs, ATP8 gene (156 bp) and NAD5 gene (1582 bp) had the shortest and longest transcript, respectively. Besides, TAA was the most termination codon in mitochondrial PCGs; and COX2, NAD3, NAD5, NAD4, NAD6 and Cob had incomplete termination codons. The transcript length of rrnS and rrnL was 731 bp and 1219 bp, respectively. The two rRNAs were located between trnL and control region and separated by trnV. The transcript length of 22 tRNAs ranged from 57 to 67 bp. In order to identify the phylogenetic relationship between *Scorpiops tibetanus* and six closely related species, we depicted a phylogenetic tree according to the complete mitochondrial genome of *Scorpiops tibetanus* and six closely related species [GenBank accession numbers: *Mesobuthus martensii* (NC-009738.1), *Mesobuthus gibbosus* (AJ716204.2), *Centruroides limpidus* (NC-006896.1), *Tityus serrulatus* (KR024030.1), *Heterometrus longimanus* (KR190462.1) and *Buthus occitanus* (NC-010765.1)]. MAFFT was used for sequence alignment among species, and then RAxML (V8.2.10) was used, GTR GAMMA model, rapid bootstrap analysis, bootstrap = 1000, MEGA7.0 maximum likelihood method was used to construct the phylogenetic tree (Kumar et al. 2016). According to the results of phylogenetic tree analysis (Figure 1), the *Scorpiops tibetanus* is sister species to *Heterometrus longimanus*, which were consistent with the traditional classification.

**Figure 1.**
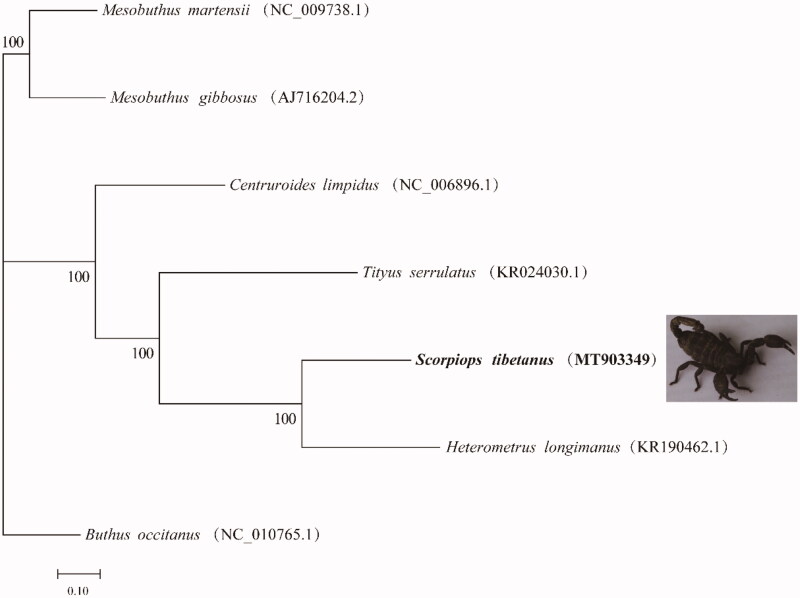
Neighbor-Joining phylogenetic tree analysis for *Scorpiops tibetanus* and six closely related species.

## Data Availability

Supporting this study are openly available in NCBI:GenBank accession number at https://www.ncbi.nlm.nih.gov/nuccore/ MT903349, SRA accession number at https://www.ncbi.nlm.nih.gov/sra/ SRR12854514.Associated accession numbers at https://www.ncbi.nlm.nih.gov/nuccore [NC-009738.1, AJ716204.2, NC-006896.1, KR024030.1, KR190462.1, NC-010765.1].
